# Threats of evictions in the USA: A public health concern

**DOI:** 10.1016/j.amsu.2022.104681

**Published:** 2022-09-15

**Authors:** Abdullahi Tunde Aborode

**Affiliations:** Mississippi State University, Starkville, USA

A good place to live is a medicine for health. However, millions of families and individuals are unable to afford a secure home as housing costs rise while incomes are stagnant in the USA [1]. Across the nation, supporting services and affordable housing are being incorporated in public health programs to combat homelessness. In America, evictions are a common occurrence among renters, and they are particularly likely to happen to women, persons of color, and families with young children [1].

Eviction, however, is a different public health issue affecting the neighborhoods in USA. The possibility of eviction affects an estimated 2.8 million families [2]. This unseen issue that lurks in the depths of local societies is brought to light by recent study on the health effects of displacement [2,6]. Even before they lose their house, people who face eviction threats are more likely to suffer physical illness, high blood pressure, despair, anxiety, and psychological anguish [2]. Eviction frequently results in housing instability, relocation to subpar housing, crowding, and homelessness, all of which are linked to ill health in both adults and children.

In 2016, the most recent year for which there are national data, 6.1% of renter households received eviction notifications, and 2.3% of those households actually had their properties removed from them [2]. Despite significant regional differences in the demographics of evicted tenants in the US, Black and Hispanic women and families with children are disproportionately at risk of eviction. Despite the fact that about 20% of renters are Black, Black tenants are targeted for eviction in over 33% of cases [3]. Women are 2% more likely than men to be evicted, and Black women are nearly twice as likely as White women to file for eviction (6.4 versus 3.4%) [3].

In 2017, more than 75% of evictions were due to unpaid rent [2]. With a median rent increase of 13% over the previous 20 years and a median income gain of less than 5%, rising rents in the US have outperformed wage growth by a significant margin [3]. In the US, more than half of poor tenants (those making less than 80% of the area median family income as determined by the Department of Housing and Urban Development) were deemed rent burdened in 2017 because they spent at least 30% of their income on housing [3]. Therefore, rather than just being a sudden financial shock, eviction frequently reflects the ongoing financial precarity of renters.

Adults who have been evicted often have bad health. Adults who have been evicted die from more causes than matched controls. Adults who have been evicted report having worse physical health than those who have not been evicted, and quasi-experimental investigations show that eviction increases the frequency of visits to the emergency room [4]. Eviction increases the likelihood that an HIV-positive person's viral load will be detectable, and it also raises the likelihood that someone may start using drugs again. Individuals who have been evicted from their homes report much lower mental health than other adults, and they also have higher suicide mortality and hospitalization rates for mental illness [4]. Many adults who have just been evicted from their homes report experiencing increased levels of despair, anxiety, and insomnia, and counties with greater eviction rates had much higher rates of unintentional drug-use [4].

Eviction is linked to numerous other socioeconomic factors that affect health, which could amplify its unfavorable effects [7]. Compared to other low-income children living in rented property, children whose families have experienced an eviction had greater rates of food insecurity and less favorable educational outcomes [4]. Many evicted families claim that their kids had to switch schools as a result of the eviction. Tenants who have been evicted are more likely than tenants who have not been evicted to move into residences of inferior quality (i.e., homes with damaged appliances, exposed cables, or insufficient heating) and experience more subsequent moves [8]. Eviction can also have disastrous financial repercussions; tenants who have been evicted are more likely than other renters to rely on welfare programs, lose their jobs, and have limited access to credit.

It is unacceptable that the number of evictions is increasing and that it disproportionately affects women of color and their children in our neighborhoods. Promoting health equity and enhancing public health across the nation require policies that lessen evictions. The COVID-19 pandemic's economic effects have recently raised the number of renters at risk of being evicted [4,8]. The pandemic's major infection prevention tactics are undermined by housing instability, which exacerbates the negative health effects of eviction. It is now more important than ever to investigate the full scale of the eviction situation, comprehend how eviction impacts health and health justice, and learn about the policy options available to fight eviction both during and after the COVID-19 pandemic [4,5].

In the areas where people want to live, learn, and work, more money has to be put into affordable housing. The health of the communities and the nation depend on stepping up efforts to increase housing availability while advancing racial fairness and specifically addressing the needs of those who are most at danger of being displaced and evicted. The health system can help decrease evictions, forced relocation, and housing instability. Health systems can take advantage of financial resources to increase housing stability by making investments in supportive services and affordable housing. Additionally, healthcare systems and organizations are starting to raise their voices in support of better housing laws and more federal and state funding.

The basis of good health is a secure household. People are healthier when they live in homes they can afford in the areas where they want to. Institutions in the healthcare and public health sectors need to do more to lessen forcible evictions and displacement. Everyone has the right to a safe, decent, and affordable place to live, and when they do, our entire community benefits from happier, more prosperous neighborhoods. The effects of eviction on people's health are expected to worsen racial and socioeconomic gaps in health since eviction generally has a disproportionately negative impact on low-income individuals of color. An extensive set of policy measures is needed to address the eviction situation. Financial support for tenants and the extension of moratoria can mitigate the pandemic's economic effects in the short run.

In order to solve the problem of poor renters losing their homes when a pandemic is raging, immediate action is necessary. The standard of living and quality health of evicted people could be enhanced and extended temporarily by halting earlier steps in the eviction procedure and by including all eviction cases, not simply those involving unpaid rent. Rental support programs must be put into place right now in order to decrease evictions and give tenants and landlords the money they need to get through the current economic and public health crises. Long-term policies must be put in place by the federal, state, and local governments to provide lower-income Americans with safe and affordable housing.

## Ethical approval

There is no ethical rule for this study.

## Source of funding

There is no funding available for this research.

## Author contribution

Abdullahi Tunde Aborode conceptualized the study, design the study, did the data collection, data analysis, data interpretation, writing of the paper, edit of the paper and final approval of the paper.

## Trail registry number

None.

## Guarantor

There is no guarantor.

## Declaration of competing interest

There is no funding available for this study.
